# Comparison between Frailty Index of Deficit Accumulation and Phenotypic Model to Predict Risk of Falls: Data from the Global Longitudinal Study of Osteoporosis in Women (GLOW) Hamilton Cohort

**DOI:** 10.1371/journal.pone.0120144

**Published:** 2015-03-12

**Authors:** Guowei Li, Lehana Thabane, George Ioannidis, Courtney Kennedy, Alexandra Papaioannou, Jonathan D. Adachi

**Affiliations:** 1 Department of Clinical Epidemiology & Biostatistics, McMaster University, Hamilton, Ontario, Canada; 2 St. Joseph’s Hospital, McMaster University, Hamilton, Ontario, Canada; 3 Department of Medicine, McMaster University, Hamilton, Ontario, Canada; Mayo Clinic, UNITED STATES

## Abstract

**Objectives:**

To compare the predictive accuracy of the frailty index (FI) of deficit accumulation and the phenotypic frailty (PF) model in predicting risks of future falls, fractures and death in women aged ≥55 years.

**Methods:**

Based on the data from the Global Longitudinal Study of Osteoporosis in Women (GLOW) 3-year Hamilton cohort (n = 3,985), we compared the predictive accuracy of the FI and PF in risks of falls, fractures and death using three strategies: (1) investigated the relationship with adverse health outcomes by increasing per one-fifth (i.e., 20%) of the FI and PF; (2) trichotomized the FI based on the overlap in the density distribution of the FI by the three groups (robust, pre-frail and frail) which were defined by the PF; (3) categorized the women according to a predicted probability function of falls during the third year of follow-up predicted by the FI. Logistic regression models were used for falls and death, while survival analyses were conducted for fractures.

**Results:**

The FI and PF agreed with each other at a good level of consensus (correlation coefficients ≥ 0.56) in all the three strategies. Both the FI and PF approaches predicted adverse health outcomes significantly. The FI quantified the risks of future falls, fractures and death more precisely than the PF. Both the FI and PF discriminated risks of adverse outcomes in multivariable models with acceptable and comparable area under the curve (AUCs) for falls (AUCs ≥ 0.68) and death (AUCs ≥ 0.79), and c-indices for fractures (c-indices ≥ 0.69) respectively.

**Conclusions:**

The FI is comparable with the PF in predicting risks of adverse health outcomes. These findings may indicate the flexibility in the choice of frailty model for the elderly in the population-based settings.

## Introduction

With an estimate of two billion people older than 60 years by 2050 [1], the accelerating population aging has caused profound concerns. Clinical conditions of frailty become the most problematic expression of population aging [2], which affects the elderly in their physical, psychological and social functioning and increases the risks of adverse health outcomes [3]. Emerging evidence shows that frail individuals have greater nonspecific vulnerability and are more susceptible to falls, fractures, institutionalization, hospitalization, disability and death, compared with the robust elderly people [2,4,5].

Currently, there are two wide-spread approaches to measuring frailty in the elderly: the phenotypic frailty (PF) model [6] and the frailty index (FI) of cumulative deficits [7]. The PF model was based on five physical indicators including unintentional weight loss, exhaustion, weakness, slow walking and low physical activity using data from the Cardiovascular Health Study (CHS) [6], while the FI used a variety of individual health deficits to quantify the cumulative effect in the cohort of the Canadian Study of Health and Aging (CSHA) [7]. Nevertheless, even with statistical convergence and overlap, the continuous FI of deficit accumulation presented higher discrimination for people with frailty than the categorical PF model [2]. E.g., some studies showed the FI approach could significantly characterize the risk of mortality for the elderly individuals, whereas the PF measure obtained non-significant results [8–10].

Falls are a significant public health problem in the elderly, with about 30% of community-dwellers and over 40% institutionalized residents aged ≥ 65 years falling every year [11,12]. Falls often lead to decline in functional capacity in the elderly and are related to increased morbidity, mortality and impaired quality of life [13–15]. Many studies have used a PF or FI approach to examine the relationship between frailty and risk of falls [6,16–18], however, none of them compared the predictive accuracy of PF and FI models for falls. Moreover, previous studies typically reported their findings for falls only in the elderly aged ≥ 65 years, evidence comparing the predictive accuracy of the PF and FI approaches in adults < 65 years is needed.

In this study, our primary objective was to compare the predictive accuracy of the FI and PF models in predicting risk of future falls in women aged ≥ 55 years, based on the data from the Global Longitudinal Study of Osteoporosis in Women (GLOW) 3-year Hamilton cohort. The secondary objective was to compare the predictive accuracy of the PF and FI approaches for fractures and death.

## Methods

### Participants and setting

The GLOW is a longitudinal cohort study involving 17 sites in 10 countries (Canada, Germany, Australia, Netherlands, Belgium, Italy, Spain, France, UK, and US). It was designed to investigate risk factors for and health consequences of fragility fractures in 60,393 women aged ≥ 55 years who had visited their physician practices in the past two years, which has been described in detail previously [19].

This study only used data in the Hamilton, Canada site. I.e., our study was a longitudinal analysis of the 3-year GLOW Hamilton cohort of women. A sample of 4,000 women approximately agreed to participate (response rate: 58%) and were enrolled between May 2008 and March 2009, based on the GLOW Hamilton cohort. The participants were stratified according to age strata to lead to two-thirds of women aged ≥ 65 years. The eligibility criteria included that the women had no cognitive impairment or language barriers, and were not institutionalized or too ill to finish the study survey. The surveys were conducted by mailing questionnaires to participants annually. The questionnaires covered the domains of participant characteristics and risk factors, medication use, health care use and access, co-morbidities, perception about fracture risk and osteoporosis, physical function, physical activity and quality of life [19]. For the non-responders during follow-up, we called the homes of those participants who did not mail back their annual questionnaire.

The study was approved by the Western Institutional Review Board. Written informed consent was obtained from all participants.

### PF model and FI approaches

The baseline PF was defined according to five criteria for unintentional weight loss, exhaustion, weakness, slow walking and low physical activity [6]. Based on the data from GLOW study, questions were chosen to create a frailty instrument similar to the Women’s Health Initiative (WHI) instrument [20] including the domains of slowness and weakness, low physical activity, poor endurance and exhaustion, and unintentional weight loss, which had been described in detail previously [21]. Briefly, for the domain of slowness and weakness, we used the Medical Outcomes Study 36-item Short Form Survey (SF-36) physical functioning component to assess the limitations in 10 activities such as running, bathing, bending, and walking [19]. In each of the activities, participants received 0 point if they reported they were limited a lot, and 50 points if they were limited a little, and 100 points if they were not limited at all respectively [21]. To measure poor endurance and exhaustion, we used the vitality component from the SF-36 including four questions on whether they felt worn out, felt full of life, felt tired, or had a lot of energy. For responses to feeling worn out and feeling tired, the scores were 100 points for none of the time, 75 points for a little of the time, 25 points for most of the time, and 0 point for all of the time. The scores were reversely coded for responses to having a lot of energy and feeling full of life [21]. We evaluated the domain of low physical activity by asking participants about the number of days that in the past 30 days they had walked at least 20 minutes. As regards unintentional weight loss, participant documented whether they had lost 10 pounds or more unintentionally in the last 12 months [19].

We adopted Wood’s scoring methodology in the WHI observational study to calculate the PF scores [20]. For the domains of endurance and exhaustion, slowness and weakness, and physical activity, assignment of points was according to a score in the lowest quartile of their respective distribution [20,21]. Specifically, to align with the separate components of slowness and weakness in the Fried’s PF from the CHS, women in the lowest quarter of slowness and weakness received 2 points [6,20,21]. Participants in the lowest quartile of poor endurance and exhaustion obtained 1 point. Women in the lowest quarter for physical activity scored 1 point, while participants reporting unintentionally losing 10 pounds or more in the past year also received 1 point [20]. Women with an aggregate PF score of 3, 4, or 5 were categorized as frail, while those with a total PF score of 1 or 2 were considered as pre-frail. Participants with a PF score of 0 were classified as robust [6,20,21].

The baseline FI consisted of 34 health deficits including symptoms and signs (n = 6), comorbidities (n = 15), activity of daily living (n = 12), and healthcare utilization (n = 1), which had also been described in detail in another paper using the GLOW Hamilton cohort [22]. Each deficit was polychotomized or dichotomized and mapped to the interval 0–1 (e.g., for the question of ‘feels full of life’: the response of ‘all the time’ was coded as 0, ‘most of time’ as 0.25, ‘some time’ as 0.5, ‘a little time’ as 0.75 and ‘none of time’ as 1) to represent the severity or frequency of the deficit [22]. The specific deficit variables and their coding could be seen in S1 Table. Then the values of deficits were added up and divided by the total number of items (n = 34), to calculate a FI for each participant. E.g., if a woman had 5 deficits with each score of 1 point, 25 deficits with each score of 0, and the other 4 deficits with each score of 0.25 point, then her accumulative scores of deficits would therefore be 6 divided by 34 giving a FI = 0.18. The FI ranged from 0 to 1 [22].

### Outcomes

In this study, since the dates for falls were not available, the primary outcome was falls during the third year of follow-up. The secondary outcomes were fractures and death during the 3-year follow-up. All the outcomes were self-reported and medical records were not available to validate the data.

For falls, women reported the number of incident falls (more than one time, one time, none) in the previous 12 months at baseline and during the follow-up on the annually mailed questionnaires [22].

Baseline fractures since 45 years old and incident fractures during the follow-up included fractures of the ankle, lower leg, upper leg, pelvis, hip, rib, spine, wrist, upper arm or clavicle, were self-reported on annual surveys. The dates of incident fractures during the follow-up were also documented on questionnaires by self-report. As regards death, some spouses and family members informed us of the participant’s death when they received survey mailings from us, or when we called the homes of the non-responders. If we could not contact the household of the participants who failed to mail back their annual questionnaires, we searched electronic databases of obituaries for entries that matched the participant’s dates of birth and full names [22].

### Statistical analyses

The three groups based on the PF classification (robust, pre-frail, frail) were reported as the number and percentages of women, while the mean and standard deviation (SD) was showed for the continuous FI. The comparison of the robust, pre-frail and frail group at baseline was assessed using the analysis of variance (ANOVA) for continuous variables and Chi-square tests for categorical variables.

To compare the predictive power of the PF and FI models directly, we chose three strategies to quantify the findings.

1. The predicted risk of adverse health outcomes was compared for the PF and FI approaches, based on the incremental predictive power. I.e., since the PF comprised 5 items with the aggregate PF scores from 0 to 5 points, we calculated the risk of outcomes by increasing per-1 point of the PF scores. On the other hand, for the FI model, the increased risk was obtained and compared by increasing per one-fifth (i.e., 20%) of the FI, where the FI ranged from 0 to 0.72 in our study. This methodology was similar to the strategy using per 1% increase both in the PF and FI to quantify the risks in one previous study [8].
2. Using Rockwood’s methodology [10], we chose the cut-points to trichotomize the FI, based on the overlap in the density distribution of the FI by the three groups (robust, pre-frail and frail) which were defined by the PF. Subsequently, the predictive accuracy of the FI model using the cut-points for adverse health outcomes was compared with that of the PF approach.
3. We firstly obtained a predicted probability function of falls during the third year of follow-up predicted by the FI, using a multivariable logistic regression model adjusted for age, smoking, drinking, body mass index (BMI), education and baseline falls. Then the predicted risk of falls during the third year of follow-up was categorized into low-, medium- and high- risk group, based on the cut-points of 0.27 and 0.50. The cut-points were chosen because the estimated annual probability of falling was 0.27 in the elderly, and the annual fall risk was 0.50 if the elderly fell in the past year or they had clinically detected abnormalities of gait or balance [23,24]. This strategy was similar to the categorized methodology based on the predicted absolute 10-year fracture risk calculated by the WHO Fracture Risk Assessment (FRAX) tool [25–28]. Accordingly, the predictive accuracy was compared between the groups (low-, medium- and high-risk) categorized by the FI and the groups (robust, pre-frail and frail) defined by the PF, taking low-risk and robust group as reference group for the FI and PF respectively.

The frequencies and proportions of participants according to the PF and FI criteria for each strategy were shown. The agreement between the FI and PF was quantified by Spearman rank correlation coefficients.

Since the dates for falls were not available, we investigated the relationship between baseline PF or FI and risk of falls only using the data on the falls during the third year of follow-up. Two binary logistic regression models were performed and compared: baseline age-adjusted model and fully-adjusted multivariable model. Multivariable binary logistic regression models were adjusted for age, education, smoking, drinking, BMI and baseline falls, to explore the association between baseline PF or FI and risk of falls with the use of odds ratio (OR). Areas under the curve (AUC) were calculated from the receiver operating characteristic curves (ROC), in which the AUC could judge the discrimination of the models. The ROC contrasts were used to compare the AUCs for the PF and FI. The Goodness-of-fit of the models was assessed using a Hosmer-Lemeshow statistic.

Because the dates were only available for incident fractures, logistic regression was conducted to analyze the relationship between baseline PF or FI and risk of death, while Cox proportional hazards regression was used for fractures. OR and hazard ratio (HR) were used to quantify the relationship for death and fractures, respectively. AUC were used to assess the discrimination of the models for death, whereas the c-index measured the predictive power for fractures [29]. The proportional hazards assumption for fractures was evaluated using both a statistical test and the Schoenfeld residuals [30], and no violations of the proportional hazards assumption were found in this study. The Goodness-of-fit of the models for fractures was assessed by the Akaike information criterion (AIC) [31].

Subgroup analyses were conducted using the cut-point of 65 years to separate the younger women (< 65 years) from the older women (≥ 65 years). With regard to missing data, if < 10% on a variable were missing, we used the median or mean of the variable in its group to impute the missing data. If ≥ 10% of data were missing, ten multiple imputations were performed by including other relevant variables which were selected by clinical knowledge [32,33]. All tests were two-sided with the level of significance at 0.05. All analyses were performed using SAS, version 9.3 (SAS Institute, Inc., Cary, NC).

## Results

There were 3,985 women at baseline in this study (Table 1). The mean of the age was 69.4 (SD: 8.89) years, and approximately one third of the women (35%) were < 65 years old. About 49% of the women drank alcohol and 11% were smokers. Most women did not have overnight hospitalization in the past year (89%), prior fractures since 45 years old (78%), or falls in the last 12 months (62%). The mean FI in the women was 0.24 (SD: 0.13).

**Table 1:** Baseline characteristics of study participants and comparison between robust, pre-frail and frail women according to the PF classification^1^.

Characteristics	Overall Participants (n = 3,985)	Robust women (n = 1,894)	Pre-frail women with (n = 1,479)	Frail women (n = 612)	P-Value
Age: mean (SD), years	69.4 (8.89)	68.2 (8.23)	69.5 (8.96)	73.2 (9.63)	<0.001^2^
Age strata, n (%)					
55–64	1,385 (34.76)	728 (38.44)	517 (34.96)	140 (22.88)	<0.001^3^
65–74	1,423 (35.71)	718 (37.91)	521 (35.23)	184 (30.07)	
75–84	952 (23.89)	387 (20.43)	352 (23.80)	213 (34.80)	
≥85	225 (5.65)	61 (3.22)	89 (6.02)	75 (12.25)	
BMI: mean (SD), kg/m^2^	27.7 (5.77)	26.7 (4.69)	28.2 (5.82)	29.7 (7.75)	<0.001^2^
Smoker, n (%)					
Yes	447 (11.3)	167 (8.87)	192 (13.06)	88 (14.55)	<0.001^4^
No	3,510 (88.70)	1715 (91.13)	1278 (86.94)	517 (85.45)	
Drinking (drinks/week), n (%)					
0	2,027 (51.21)	832 (44.16)	770 (52.45)	425 (70.13)	<0.001^3^
<7	1,414 (35.73)	756 (40.13)	521 (35.49)	137 (22.61)	
7–13	428 (10.81)	248 (13.16)	147 (10.01)	33 (5.45)	
≥14	89 (2.25)	48 (2.55)	30 (2.04)	11 (1.82)	
Race, n (%)					
White	3,717 (93.27)	1769 (93.40)	1382 (93.44)	566 (92.48)	0.70 ^4^
Non-white	268 (6.73)	125 (6.60)	97 (6.56)	46 (7.52)	
Education, n (%)					
High school or less	2,509 (64.10)	1099 (59.15)	973 (66.83)	437 (72.83)	<0.001^4^
More than high school	1,405 (35.90)	759 (40.85)	483 (33.17)	163 (27.17)	
Prior fractures since 45 years old, n (%)					
Yes	862 (22.31)	345 (18.68)	343 (23.97)	174 (29.74)	<0.001^4^
No	3,001 (77.69)	1502 (81.32)	1088 (76.03)	411 (70.26)	
Overnight hospitalization in last 12 months, n (%)					
0	3,498 (88.65)	1739 (92.40)	1293 (88.56)	466 (77.15)	<0.001^3^
1	337 (8.54)	123 (6.54)	125 (8.56)	89 (14.74)	
≥2	111 (2.81)	20 (1.06)	42 (2.88)	49 (8.11)	
Falls in last 12 months, n (%)					
0	2471 (62.49)	1237 (65.76)	933 (63.56)	301 (49.75)	<0.001^3^
1	853 (21.57)	420 (22.33)	299 (20.37)	134 (22.15)	
≥2	630 (15.93)	224 (11.91)	236 (16.08)	170 (28.10)	
Frailty index: mean (SD)	0.24 (0.13)	0.17 (0.09)	0.26 (0.11)	0.43 (0.10)	<0.001^2^

^1^ Mean follow-up = 3.01 years;

^2^ One-way analysis of variance (ANOVA) test;

^3^ Mantel-Haenszel Chi-square test;

^4^ Chi-square test;

PF: phenotypic frailty; SD: standard deviation; BMI: body mass index

Based on the PF classification criteria [21], there were 1,894 (48%), 1,479 (37%) and 612 (15%) women categorized as robust, pre-frail and frail respectively (Table 1). There was significant difference between age, BMI, proportions of smokers and alcohol drinkers, education, previous fractures, overnight hospitalization and prior falls in these three groups (P < 0.001). Also the FI was significantly different in the robust (FI = 0.17), pre-frail (FI = 0.26) and frail (FI = 0.43) women as defined by the PF approach (P < 0.001).

The frequencies and proportions of participants using the three strategies are demonstrated in Table 2. The cut-points of the FI were 0.14, 0.28, 0.42 and 0.56 for strategy 1, while the cut-points were 0.20 and 0.35 for strategy 2. Regarding strategy 3, the mean (SD) of the FI was 0.18 (0.09) for low-risk, 0.29 (0.13) for medium-risk and 0.35 (0.15) for high-risk group respectively. The PF and FI were significantly correlated with each other, with the Spearman rank correlation coefficients of 0.62, 0.56 and 0.56 for the strategy 1, 2 and 3 respectively (P < 0.001).

**Table 2:** Frequencies and proportions of participants according to PF and FI criteria using strategy 1, 2 and 3.

Strategy	Number of participants (%)	
	PF	FI
**Strategy 1** ^**1**^		
0	1894 (47.53)	3 (0.08)
1	958 (24.04)	1009 (25.32)
2	521 (13.07)	1563 (39.22)
3	470 (11.79)	965 (24.22)
4	133 (3.34)	376 (9.44)
5	9 (0.23)	69 (1.73)
**Strategy 2** ^**2**^		
Robust	1894 (47.53)	1749 (43.89)
Pre-frail	1479 (37.11)	1357 (34.05)
Frail	612 (15.36)	879 (22.06)
**Strategy 3** ^**3**^		
Robust (low-risk)	1894 (47.53)	2014 (50.54)
Pre-frail (medium-risk)	1479 (37.11)	1277 (32.05)
Frail (high-risk)	612 (15.36)	694 (17.42)

^1^ For FI, the cutpoints are 0.14, 0.28, 0.42 and 0.56; Spearman rank correlation coefficient between PF and FI: 0.62 (95% CI: 0.60–0.64), p<0.001;

^2^ For FI, the cutpoints are 0.20 and 0.35; Spearman rank correlation coefficient between PF and FI: 0.56 (95% CI: 0.54–0.58), p<0.001;

^3^ Robust, pre-frail and frail group for the PF; low-, medium- and high-risk group for the FI; the mean (standard deviation) FI: 0.18 (0.09) for low-risk, 0.29 (0.13) for medium-risk and 0.35 (0.15) for high-risk group; Spearman rank correlation coefficient between PF and FI: 0.56 (95% CI: 0.54–0.59), p<0.001

PF: phenotypic frailty; FI: frailty index

The comparison of the associations between the PF and FI and falls, fractures and death using strategy 1 is shown in Table 3. During the third year of follow-up, 32% (n = 1,068) reported at least one fall in the previous year, while 6.36% (n = 238) reported incident fractures and 2.69% (n = 107) women died during the 3-year follow-up. Results of multivariable logistic regression showed that the associations between the per-20% incremental FI and PF and risk of falls were significant (OR = 1.37, 95% confidence interval (CI): 1.25–1.50 for FI; OR = 1.15, 95% CI: 1.07–1.23 for PF). Significant relationship was also found for fractures (HR = 1.25 and 1.14 for the FI and PF respectively) and death (OR = 1.81 and 1.46 for the FI and PF respectively) (Table 3). Subgroup analyses yielded similar results in older women and younger women aged less than 65 years, and the statistics for falls, fractures and death between the subgroup were not significantly different (P > 0.05). However, AUC comparison for the FI and PF indicated that the FI (AUC = 0.80) was more predictive than PF (AUC = 0.79) in only death (P = 0.04), whereas the AUCs for falls (AUC = 0.69 and 0.68 for FI and PF respectively; P = 0.06) or the c-indices for fractures (c-index = 0.69 and 0.71 for FI and PF respectively; P = 0.59) were not significantly different (Table 4). Similar results of Goodness-of-fit tests were observed, and the statistics implied that the models for PF and FI were good calibration (S2 Table).

**Table 3:** Comparison of relationship between PF/FI approaches and falls, fractures and death using strategy 1*.

Outcomes	Strategy 1^1^			
	Age-adjusted model		Multivariable model^2^	
	PF	FI	PF	FI
**Falls (n = 1,068, 31.89%)**	**1.24 (1.16–1.32)**	**1.51 (1.39–1.63)**	**1.15 (1.07–1.23)**	**1.37 (1.25–1.50)**
Women<65 yr	1.25 (1.12–1.40)	1.51 (1.32–1.71)	1.12 (0.98–1.28)^3^	1.28 (1.09–1.51)
Women ≥65 yr	1.22 (1.13–1.32)	1.50 (1.36–1.66)	1.16 (1.07–1.27)	1.41 (1.26–1.58)
**Fractures (n = 238, 6.36%)**	**1.16 (1.04–1.28)**	**1.31 (1.15–1.48)**	**1.14 (1.01–1.28)**	**1.25 (1.08–1.45)**
Women<65 yr	1.23 (1.01–1.49)	1.27 (1.01–1.60)	1.27 (1.00–1.61) ^4^	1.32 (1.00–1.74)^4^
Women ≥65 yr	1.13 (1.00–1.27) ^4^	1.32 (1.13–1.54)	1.11 (0.96–1.27) ^3^	1.21 (1.02–1.44)
**Death (n = 107, 2.69%)**	**1.47 (1.27–1.70)**	**1.78 (1.46–2.16)**	**1.46 (1.25–1.69)**	**1.81 (1.46–2.24)**
Women<65 yr	1.58 (1.04–2.39)	1.71 (1.01–2.90)	1.84 (1.17–2.91)	2.00 (1.12–3.58)
Women ≥65 yr	1.45 (1.24–1.70)	1.79 (1.45–2.21)	1.42 (1.21–1.67)	1.79 (1.42–2.25)

* Results were expressed as statistics (95% CI); PF: phenotypic frailty; FI: frailty index;

^1^ Risk of adverse outcomes was measured on the basis of per 20% (1/5) increase of the PFS or FI; For FI, an increase was a change of 0.14 on the FI;

^2^ Multivariable model adjusted for age, smoking, drinking, BMI, education and baseline falls for falls; adjusted for age, smoking, drinking, baseline fracture, family history of fractures, BMI and education for fractures; adjusted for age, smoking, drinking, BMI and education for death;

^3^ P-value>0.05;

^4^ P-value = 0.05

**Table 4:** Comparison of predictive accuracy between PF and FI approaches in predicting falls, fractures and death*.

Outcomes	Statistics^1^ for strategy 1		Statistics^1^ for strategy 2		Statistics^1^ for strategy 3	
	Age-adjusted model	Multivariable model ^2^	Age-adjusted model	Multivariable model ^2^	Age-adjusted model	Multivariable model ^2^
**Falls**						
PF model	0.56 (0.54–0.58)	0.68 (0.66–0.70)	0.56 (0.54–0.58)	0.68 (0.66–0.70)	0.56 (0.54–0.58)	0.68 (0.66–0.70)
FI model	0.60 (0.58–0.62)	0.69 (0.67–0.71)	0.59 (0.57–0.61)	0.69 (0.67–0.71)	0.59 (0.57–0.61)	0.69 (0.67–0.71)
P-value for difference of AUCs	P<0.001	0.064	0.012	0.066	0.020	0.11
**Fractures**						
PF model	0.77 (0.65–0.87)	0.71 (0.59–0.82)	0.77 (0.65–0.87)	0.71 (0.59–0.82)	0.77 (0.65–0.87)	0.71 (0.59–0.82)
FI model	0.74 (0.62–0.85)	0.69 (0.57–0.80)	0.75 (0.63–0.85)	0.70 (0.57–0.81)	0.75 (0.63–0.85)	0.69 (0.57–0.81)
P-value for difference of c-indices	0.64	0.59	0.60	0.55	0.60	0.59
**Death**						
PF model	0.78 (0.74–0.83)	0.79 (0.74–0.84)	0.78 (0.73–0.82)	0.79 (0.74–0.83)	0.78 (0.73–0.82)	0.79 (0.74–0.83)
FI model	0.79 (0.75–0.84)	0.80 (0.76–0.85)	0.79 (0.74–0.83)	0.80 (0.75–0.84)	0.79 (0.75–0.83)	0.80 (0.75–0.84)
P-value for difference of AUCs	0.13	0.040	0.39	0.31	0.37	0.25

* Results were expressed as statistics (95% CI); PF: phenotypic frailty; FI: frailty index;

^1^ AUC for falls and death, and c-index for fractures;

^2^ Multivariable model adjusted for age, smoking, drinking, BMI, education and baseline falls for falls; adjusted for age, smoking, drinking, baseline fracture, family history of fractures, BMI and education for fractures; adjusted for age, smoking, drinking, BMI and education for death.

The results of relationship between the PF and FI and falls, fractures and death using strategy 2 and 3 are showed in Table 5. Multivariable analyses presented non-significant associations between the PF and outcomes in the pre-frail women (OR = 1.16, 95% CI: 0.98–1.38 for falls; HR = 1.16, 95% CI: 0.87–1.56 for fractures; OR = 1.62, 95% CI: 0.96–2.73 for death), compared to the robust women. However, significant results were found in the frail women using the PF approach: OR = 1.58 for falls; HR = 1.66 for fractures; OR = 3.51 for death. For the FI approach in the strategy 2 and 3, significant relationship between falls and the FI was observed in both the pre-frail (or medium-risk) and frail (or high-risk) women: for strategy 2, OR = 1.20 and 1.91 in the pre-frail and frail women respectively; for strategy 3, OR = 1.24 and 2.12 in the pre-frail and frail women respectively. For fractures and death, the FI approach yielded similar results using strategy 2 and 3 (for strategy 2 in the pre-frail and frail women respectively: HR = 1.33 and 1.80 for fractures, OR = 1.95 and 4.26 for death; for strategy 3 in the medium-risk and high-risk women respectively: HR = 1.39 and 1.95 for fractures, OR = 2.47 and 4.87 for death), compared to the robust (or low-risk) participants. Nevertheless, ROC contrasts showed no significant differences for falls and death between the PF and FI approaches (for strategy 2 in the PF and FI respectively: AUC = 0.68 and 0.69, P = 0.066 for falls, AUC = 0.79 and 0.80, P = 0.31 for death; for strategy 3 in the PF and FI respectively: AUC = 0.68 and 0.69, P = 0.11 for falls, AUC = 0.79 and 0.80, P = 0.25 for death) (Table 4). Moreover, c-indices indicated that the FI was not superior to the PF in predicting fractures: c-index = 0.71 and 0.70 for PF and FI respectively in strategy 2, P = 0.55; c-index = 0.71 and 0.69 for PF and FI respectively in strategy 3, P = 0.59 (Table 4). Goodness-of-fit tests implied that both the models for PF and FI were good calibration (S2 Table) for strategy 2 and 3, respectively.

**Table 5:** Comparison of relationship between the PF/FI approaches and falls, fractures and death using strategy 2 and 3*.

Outcomes	PF		FI			
	Age-adjusted model	Multivariable model^3^	Strategy 2^1^		Strategy 3^2^	
			Age-adjusted model	Multivariable model^3^	Age-adjusted model	Multivariable model^3^
**Falls (n = 1,068, 31.89%)** ^**6**^						
**Pre-frail (medium-risk)**	**1.21 (1.03–1.42)**	**1.16 (0.98–1.38)** ^**4**^	**1.34 (1.13–1.59)**	**1.20 (1.01–1.44)**	**1.40 (1.19–1.65)**	**1.24 (1.05–1.49)**
Women<65 years	1.15 (0.89–1.49) ^4^	1.07 (0.81–1.42) ^4^	1.51 (1.15–1.99)	1.28 (0.94–1.74) ^4^	1.56 (1.19–2.03)	1.30 (0.96–1.75) ^4^
Women ≥65 years	1.23 (1.00–1.51) ^5^	1.21 (0.98–1.50) ^4^	1.23 (0.99–1.53) ^4^	1.15 (0.92–1.45) ^4^	1.31 (1.06–1.61)	1.22 (0.98–1.51) ^4^
**Frail (high-risk)**	**2.03 (1.63–2.53)**	**1.58 (1.24–2.01)**	**2.55 (2.10–3.11)**	**1.91 (1.53–2.38)**	**2.98 (2.39–3.73)**	**2.12 (1.65–2.73)**
Women<65 years	2.22 (1.48–3.33)	1.48 (0.92–2.39) ^4^	2.87 (2.03–4.07)	1.86 (1.23–2.81)	3.11 (2.05–4.71)	1.91 (1.17–3.12)
Women ≥65 years	1.93 (1.48–2.50)	1.62 (1.22–2.16)	2.34 (1.84–2.98)	1.92 (1.47–2.50)	2.82 (2.16–3.70)	2.21 (1.65–2.97)
**Fractures (n = 238, 6.36%)** ^**6**^						
**Pre-frail (medium-risk)**	**1.19 (0.89–1.56)** ^**4**^	**1.16 (0.87–1.56)** ^**4**^	**1.34 (0.98–1.82)** ^**4**^	**1.33 (0.97–1.82)** ^**4**^	**1.41 (1.05–1.89)**	**1.39 (1.04–1.88)**
Women<65 years	1.63 (0.99–2.70) ^4^	1.65 (0.99–2.77) ^4^	1.42 (0.83–2.44) ^4^	1.57 (0.90–2.73) ^4^	1.83 (1.10–3.02)	2.00 (1.19–3.39)
Women ≥65 years	1.01 (0.71–1.44) ^4^	1.00 (0.70–1.43) ^4^	1.28 (0.88–1.87) ^4^	1.26 (0.86–1.85) ^4^	1.23 (0.86–1.76) ^4^	1.21 (0.84–1.74) ^4^
**Frail (high-risk)**	**1.65 (1.17–2.34)**	**1.66 (1.16–2.38)**	**1.82 (1.31–2.53)**	**1.80 (1.27–2.55)**	**1.98 (1.40–2.82)**	**1.95 (1.34–2.82)**
Women<65 years	2.05 (1.00–4.20) ^5^	2.32 (1.06–5.06)	2.20 (1.21–4.01)	2.48 (1.27–4.84)	2.24 (1.10–4.56)	2.61 (1.19–5.70)
Women ≥65 years	1.49 (1.00–2.21) ^5^	1.49 (1.00–2.24) ^5^	1.68 (1.13–2.49)	1.61 (1.07–2.44)	1.81 (1.20–2.73)	1.71 (1.12–2.62)
**Death (n = 107, 2.69%)** ^**6**^						
**Pre-frail (medium-risk)**	**1.64 (0.98–2.76)** ^**4**^	**1.62 (0.96–2.73)** ^**4**^	**1.95 (1.06–3.59)**	**1.95 (1.06–3.61)**	**2.46 (1.39–4.36)**	**2.47 (1.38–4.41)**
Women<65 years	1.45 (0.36–5.81) ^4^	1.73 (0.42–7.11) ^4^	2.36 (0.63–8.86) ^4^	3.19 (0.81–12.63) ^4^	2.29 (0.70–7.56) ^4^	2.94 (0.83–10.38) ^4^
Women ≥65 years	1.66 (0.95–2.91) ^4^	1.62 (0.92–2.84) ^4^	1.93 (0.97–3.84) ^4^	1.88 (0.94–3.76) ^4^	2.65 (1.37–5.12)	2.59 (1.33–5.04)
**Frail (high-risk)**	**3.62 (2.16–6.07)**	**3.51 (2.06–5.96)**	**4.24 (2.37–7.58)**	**4.26 (2.34–7.76)**	**4.78 (2.65–8.63)**	**4.87 (2.64–8.95)**
Women<65 years	5.44 (1.34–22.06)	9.11 (1.99–41.73)	2.96 (0.66–13.33) ^4^	4.75 (0.93–24.29) ^4^	1.26 (0.15–10.84) ^4^	1.95 (0.21–18.48) ^4^
Women ≥65 years	3.42 (1.96–5.96)	3.19 (1.81–5.63)	4.43 (2.31–8.48)	4.25 (2.19–8.26)	5.52 (2.83–10.77)	5.38 (2.71–10.68)

* Results were expressed as statistics (95% CI); PF: phenotypic frailty; FI: frailty index;

^1^ The cut-points were chosen based on Rockwood’s methodology;

^2^ The groups (low-, medium-, high-risk) were categorized according to a predicted probability function of falls during the third year of follow-up predicted by the FI;

^3^ Multivariable model adjusted for age, smoking, drinking, BMI, education and baseline falls for falls; adjusted for age, smoking, drinking, baseline fracture, family history of fractures, BMI and education for fractures; adjusted for age, smoking, drinking, BMI and education for death;

^4^ P-value>0.05;

^5^ P-value = 0.05

^6^ For strategy 2: robust, pre-frail and frail group for both the PF and FI; robust group taken as reference group; For strategy 3: robust, pre-frail and frail group for the PF; low-, medium- and high-risk group for the FI; robust and low-risk group taken as reference group for the PF and FI respectively.

Similar results were also reported consistently in the age-adjusted models and subgroup analyses for the FI and PF approaches using the three strategies (Tables 3, 4 and 5).

For the response in this cohort, there were 636 women (16.0%) with unknown status of falls during the third year of follow-up, and 244 women (6.1%) with unknown fractures during the 3-year follow-up (Table 3 and 5). A post-hoc analysis was conducted to compare their baseline age, FI and PF scores between the responders and non-responders during the follow-up. I.e., we compared the age, FI and PF scores between 3349 versus 636 women for falls, and 3741 versus 244 women for fractures, respectively. However, no significant difference was observed (all p-values > 0.05), indicating the characteristics of the non-responders during follow-up were similar to those responders used for analysis.

## Discussion

Using the GLOW Hamilton 3-year cohort, we compared the predictive accuracy of the FI and PF approaches in predicting risks of future falls, fractures and death for the elderly women. We found that increasing levels of frailty, as assessed by the FI and PF using three different strategies, were significantly related with increased risks of falls, fractures and death. The FI evaluated the chances of adverse health outcomes more precisely with higher statistics than the PF. However, most of the predictive accuracy did not differ in the two frailty measures significantly.

Some previous studies comparing the multiple deficit approach with clinical frailty criteria have found that the FI was a better predictor of frailty-related adverse health outcomes [8–10,34–37]. In our study, except mortality using strategy 1, the better accuracy of the FI was not observed in predicting outcomes (Table 4), even though the FI quantified the risks of future falls, fractures and death more precisely than the PF (Tables 3 and 5). However, results from a Chinese study [38] and the European Male Aging Study [39] indicated that the FI and PF approaches were comparable in predicting adverse health outcomes, which was in line with our findings. Furthermore, both the FI and PF discriminated adverse health outcomes with acceptable AUCs or c-indices (Table 4), and they agreed with each other at a good level of consensus (correlation coefficients ≥ 0.56, Table 2). All these findings may indicate the flexibility in the choice of frailty model in the population-based settings [38–40].

Nevertheless, the PF and FI, as the dominant tools to measure frailty in the elderly, shared conceptual differences. The PF assumed that the components of the criteria (unintentional weight loss, exhaustion, weakness, slow walking and low physical activity) were statistically independent [6], while the FI assumed that it was the accumulation of individual deficits that determined frailty [9,41]. The PF approach was simple and easy to apply in clinical settings [38], but evidence showed that it did not seize all the information needed and did not capture grades of frailty [9,42]. On the other hand, although the use of FI was not yet routine, especially the amount of information seemed daunting to non-geriatricians, a FI can be used as a population indication in evaluating the preventive or therapeutic performance of health and social services provision and policies [7,38,40,41]. Moreover, the FI may be more appropriate as a research tool given its accuracy in predicting adverse health outcomes (Tables 3, 4 and 5) and the smaller sample size required, since it covered a wider and more comprehensive range of variables than the PF including activities of daily living, co-morbidities, signs and symptoms and healthcare utilization associated with aging (S1 Table) [22].

Our data should be interpreted with caution. Although the predictive validity of the PF used in this study had been justified [21], we did not include the exact variables that the CHS proposed by Fried et al [6] to construct the PF. For instance, no timed walk or grip strength measures were available in the GLOW [19]. Instead we used the SF-36 physical functioning component to evaluate the domain of slowness and weakness. Even though the physical functioning scale had been justified as a validated surrogate for the grip strength and timed walk measures [20], the variation in the components of the PF may influence the estimates of the relationship with the adverse health outcomes to some extent, whereas the FI approach could yield comparable estimates using different variables or different number of deficits to form the FI [10,43]. The data was collected only by patient self-report, while medical records were not available to validate the data in the GLOW study [19], even though evidence showed that self-reported data were reasonably credible for adverse health outcomes in different populations and settings [44,45]. Nevertheless, self-report in longitudinal studies assisted with efficiency of data collection and methodological consistency, especially when the sample size was very large [46]. Moreover, we compared our self-report outcomes with another longitudinal study named Canadian Multicentre Osteoporosis Study (CaMos) [47,48]. The CaMos including 5,143 postmenopausal community-dwelling women reported 314 (6.11%) participants sustaining a clinically recognized incident fracture during a 3-year follow-up, in which the confirmation and information of fractures was gathered using the combination of interviews, dual-energy X-ray, questionnaires and medical treatment [47]. The incident fracture rate during the 3-year follow-up was similar to that from our study by self-report (6.36%, Tables 3 and 5), which may support the accuracy of the data from self-report in this study. Besides, the recall bias when participants were answering questionnaires could not be avoided or quantifiable. Furthermore, the population in the GLOW only consisted of women, and therefore our results may not be generalizable to elderly males.

There are some strengths of this study. Both the construction procedures of the FI and PF had been described in detail in previous studies using the GLOW data, and their predictive validity was corroborated by the significant relationship with adverse health outcomes [21,22]. The predictive power of the FI and PF was compared directly using different strategies with similar results, which justified the robustness of the findings. Moreover, this study added some value to the existing evidence on the comparison of predictive accuracy in predicting falls using the FI and PF, because previous studies typically compare the predictive validity for death or disability [8–10,35,36,38,39]. Other strengths of this study were the large sample size and the representative sample due to the unique sampling method in the GLOW study [19]. The participants over a broad age range were enrolled with few exclusion criteria and they were sampled according to the lists provided by their physician practices, which would result in the overall women being representative of the practices in real world [49].

## Conclusion

In conclusion, in this study the FI is comparable with the PF in predicting risks of future falls, fractures and death. The PF approach is simple to apply in clinical settings, while the FI may be more appropriate as a research tool. The FI and PF agree with each other at a good level of consensus and both of them predict and discriminate risks of adverse health outcomes significantly in the elderly, which may indicate the flexibility in the choice of frailty model in the population-based settings.

## Supporting Information

S1 Table The deficit variables and their coding in the FI. (DOCX)

S2 Table Comparison of the Goodness of fit statistics of models between the PF and FI approaches. (DOCX)
