# Supplementary material for: Comparison between Frailty Index of Deficit Accumulation and Phenotypic Model to Predict Risk of Falls: Data from the Global Longitudinal Study of Osteoporosis in Women (GLOW) Hamilton Cohort
Source: PLoS One. 2015 Mar 12;10(3):e0120144. doi: 10.1371/journal.pone.0120144 (PMC4357575; doi:10.1371/journal.pone.0120144)
Supplement: S1 Table — (DOCX) [file pone.0120144.s001.docx]

**Supporting Information Table S1. The deficit variables and their coding in the FI***

| **Deficit variables** | **Coding** |
| --- | --- |
| **Co-morbidities (n=15)** | |
| Taking/taken five or more medications | Yes=1, no=0 |
| Has chronic bronchitis or emphysema | Yes=1, no=0 |
| Has osteoarthritis or degenerative joint disease | Yes=1, no=0 |
| Has rheumatoid arthritis | Yes=1, no=0 |
| Suffers from stroke | Yes=1, no=0 |
| Has ulcerative colitis or Crohn’s disease | Yes=1, no=0 |
| Has celiac disease | Yes=1, no=0 |
| Has Parkinson’s disease | Yes=1, no=0 |
| Has multiple sclerosis | Yes=1, no=0 |
| Has cancer | Yes=1, no=0 |
| Has diabetes (type-1) | Yes=1, no=0 |
| Has hypertension | Yes=1, no=0 |
| Has heart disease | Yes=1, no=0 |
| Has high cholesterol | Yes=1, no=0 |
| Self rating of health | Excellent=0,very good=0.25, good=0.5, fair=0.75, poor=1 |
| **Activities of daily living (ADL) (n=12)** | |
| Limitations in vigorous activities | Yes=1, a little=0.5, no=0 |
| Limitations in moderate activities | Yes=1, a little=0.5, no=0 |
| Limitations in lifting or carrying groceries | Yes=1, a little=0.5, no=0 |
| Limitations in climbing one flight of stairs | Yes=1, a little=0.5, no=0 |
| Limitations bending, kneeling or stooping | Yes=1, a little=0.5, no=0 |
| Limitations walking one hundred yards | Yes=1, a little=0.5, no=0 |
| Limitations in bathing or dressing yourself | Yes=1, a little=0.5, no=0 |
| Needs arms to help stand up from a chair | Yes=1, no=0 |
| Number of days to walk at least 20 minutes in the past 30 days | ≤2 days=1, >2days=0 |
| Self rating of mobility | Unable=1, some problem=0.5, no problem=0 |
| Self rating of self-care | Unable=1, some problem=0.5, no problem=0 |
| Self rating of usual activities | Unable=1, some problem=0.5, no problem=0 |
| **Symptoms and signs (n=6)** | |
| Feels full of life | All the time=0, most of time=0.25, some time=0.5, a little time=0.75, none of time=1 |
| Has a lot of energy | All the time=0, most of time=0.25, some time=0.5, a little time=0.75, none of time=1 |
| Feels worn out | All the time=1, most of time=0.75, some time=0.5, a little time=0.25, none of time=0 |
| Feels tired | All the time=1, most of time=0.75, some time=0.5, a little time=0.25, none of time=0 |
| Self rating of pain/discomfort | Extremely=1, moderate=0.5, no=0 |
| Unintentional weight loss of 10 pounds | Yes=1, no=0 |
| **Healthcare utilization (n=1)** | |
| Times of visiting a healthcare provider to get medical care in the past year | None=0, 1-2 times=0.33, 3-5 times=0.67, 6 or more times=1 |

*Table from “Frailty index of deficit accumulation and falls: data from the Global Longitudinal Study of Osteoporosis in Women (GLOW) Hamilton cohort” by G Li, et al. 2014 *BMC Musculoskeletal Disorders*, 15:185. Cited with permission.
