# Supplementary material for: Comparison between Frailty Index of Deficit Accumulation and Phenotypic Model to Predict Risk of Falls: Data from the Global Longitudinal Study of Osteoporosis in Women (GLOW) Hamilton Cohort
Source: PLoS One. 2015 Mar 12;10(3):e0120144. doi: 10.1371/journal.pone.0120144 (PMC4357575; doi:10.1371/journal.pone.0120144)
Supplement: S2 Table — (DOCX) [file pone.0120144.s002.docx]

**Supporting Information Table S2. Comparison of the Goodness of fit statistics of models between PF and FI approaches**

| **Goodness of fit^1^** | **Strategy 1** | | | **Strategy 2** | | **Strategy 3** | |
| --- | --- | --- | --- | --- | --- | --- | --- |
|  | **Age-adjusted model** | | **Multivariable model ^4^** | **Age-adjusted model** | **Multivariable model ^4^** | **Age-adjusted model** | **Multivariable model ^4^** |
| **Falls^2^** | | | | | | | |
| PF model | | 7.40 (0.49) | 9.34 (0.31) | 13.02 (0.11) | 18.38 (0.019) | 13.02 (0.11) | 18.38 (0.019) |
| FI model | | 7.91 (0.44) | 14.51 (0.070) | 10.19 (0.25) | 5.78 (0.67) | 11.14 (0.19) | 4.98 (0.76) |
| **Fractures^3^** | | | | | | | |
| PF model | | 3848 | 3834 | 3850 | 3836 | 3850 | 3836 |
| FI model | | 3840 | 3827 | 3845 | 3830 | 3843 | 3831 |
| **Death^2^** | | | | | | | |
| PF model | | 4.99 (0.76) | 2.95 (0.94) | 3.52 (0.90) | 2.73 (0.95) | 3.52 (0.90) | 2.73 (0.95) |
| FI model | | 12.59 (0.13) | 7.17 (0.52) | 14.20 (0.077) | 3.59 (0.89) | 16.80 (0.032) | 8.93 (0.35) |

^1^ Hosmer-Lemeshow statistics for falls and death, and Akaike information criterion (AIC) for fractures;

^2^ Results expressed in: Hosmer-Lemeshow statistics (p-value);

^3^ Results expressed in: AIC;

^4^ Multivariable model adjusted for age, smoking, drinking, BMI, education and baseline falls for falls; adjusted for age, smoking, drinking, baseline fracture, family history of fractures, BMI and education for fractures; adjusted for age, smoking, drinking, BMI and education for death.

PF: phenotypic frailty; FI: frailty index
